# Safety and feasibility of early discharge after transcatheter aortic valve implantation with ACURATE Neo—the POLESTAR trial

**DOI:** 10.1007/s00392-024-02436-z

**Published:** 2024-04-15

**Authors:** Joris F. Ooms, Kristoff Cornelis, Harindra C. Wijeysundera, Bert Vandeloo, Jan Van Der Heyden, Jan Kovac, David Wood, Albert Chan, Joanna Wykrzykowska, Liesbeth Rosseel, Michael Cunnington, Frank van der Kley, Benno Rensing, Michiel Voskuil, David Hildick-Smith, Nicolas M. Van Mieghem

**Affiliations:** 1 Department of Interventional Cardiology, Thoraxcenter, Erasmus University Medical Center, Rotterdam, The Netherlands; 2 AZ Maria Middelares, Ghent, Belgium; 3 Sunnybrook Health Sciences Centre, Toronto, ON Canada; 4 University Hospital Brussels, Brussels, Belgium; 5 Sint Jan Hospital, Brugge, Belgium; 6 University Hospitals Leicester NHS Trust, Leicester, UK; 7 Centre for Heart Valve Innovation, St. Paul’s Hospital, University of British Columbia, Vancouver, BC Canada; 8 Royal Columbian Hospital, New Westminster, BC Canada; 9 University Medical Center Groningen, Groningen, The Netherlands; 10 ASZ Aalst, Aalst, Belgium; 11 Leeds Teaching Hospitals NHS Trust, Leeds, UK; 12 Leiden University Medical Center, Leiden, The Netherlands; 13 Sint Antonius Hospital, Nieuwegein, The Netherlands; 14 University Medical Center Utrecht, Utrecht, The Netherlands; 15 University Hospitals Brighton and Sussex NHS Trust, Brighton, UK

**Keywords:** TAVI, Self-expanding valve, Early discharge

## Abstract

**Background:**

Transcatheter aortic valve implantation (TAVI) serves a growing range of patients with severe aortic stenosis (AS). TAVI has evolved to a streamlined procedure minimizing length of hospital stay.

**Aims:**

To evaluate the safety and efficacy of an early discharge (ED) strategy after TAVI.

**Methods:**

We performed an international, multi-center, prospective observational single-arm study in AS patients undergoing TAVI with the ACURATE valve platform. Eligibility for ED was assessed prior to TAVI and based on prespecified selection criteria. Discharge ≤ 48 h was defined as ED. Primary Valve Academic Research Consortium (VARC)-3-defined 30-day safety and efficacy composite endpoints were landmarked at 48 h and compared between ED and non-ED groups.

**Results:**

A total of 252 patients were included. The median age was 82 [25th–75th percentile, 78–85] years and the median Society of Thoracic Surgeons Predicted Risk of Mortality (STS-PROM) score was 2.2% [25th–75th percentile, 1.6–3.3]. ED and non-ED were achieved in 173 (69%) and 79 (31%) patients respectively. Monitoring for conduction disturbances was the principal reason for non-ED (33%). Overall, at 30 days, all-cause mortality was 1%, new permanent pacemaker rate was 4%, and valve- or procedure-related rehospitalization was 4%. There was no difference in the primary safety and efficacy endpoint between the ED and non-ED cohorts (OR 0.84 [25th–75th percentile, 0.31–2.26], *p* = 0.73, and OR 0.97 [25th–75th percentile, 0.46–2.06], *p* = 0.94). The need for rehospitalization was similarly low for ED and non-ED groups.

**Conclusion:**

Early discharge after TAVI with the ACURATE valve is safe and feasible in selected patients. Rhythm monitoring and extended clinical observation protracted hospital stay.

**Graphical Abstract:**

Safety and feasibility of early discharge after transcatheter aortic valve implantation with ACURATE Neo, an international multi-center, prospective observational single-arm study. OR, odds ratio (95% confidence interval); VARC, Valve Academic Research Consortium

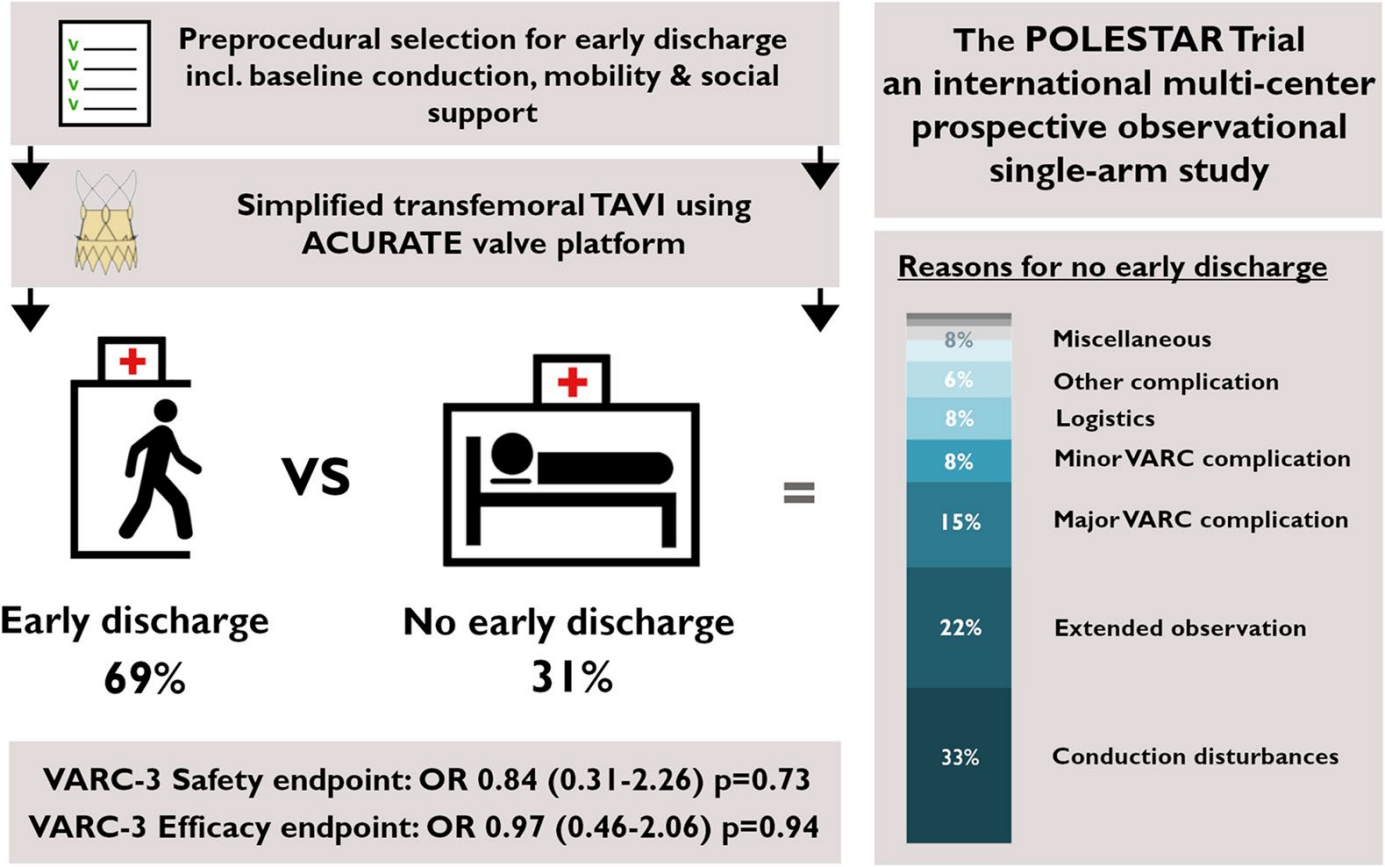

**Supplementary Information:**

The online version contains supplementary material available at 10.1007/s00392-024-02436-z.

## Introduction

Transcatheter aortic valve implantation (TAVI) for severe aortic stenosis (AS) has evolved since its conception in the beginning of the century [1]. Transcatheter heart valve (THV) iterations and operator experience drastically and reproducibly reduced serious adverse events [2–5]. A simplified lean TAVI procedure may enable early ambulation and shorter admission times [6–8]. Early hospital discharge (ED) may increase hospital capacity and reduce healthcare costs.

There is wide institutional and geographical variability in length of hospital stay (LOS) after TAVI. Comorbidities, frailty status, procedure-specific features (general anesthesia, access strategy), complications (vascular complications, neurological events, conduction disorders), and psychosocial circumstances affect LOS [9]. Patient selection and efforts to mitigate procedure complications are key for early discharge. Recent prospective studies established the safety and feasibility of an early discharge policy in selected patients after successful TAVI (FAST-TAVI) [10] or with extensive life expectancy inclusion criteria (3 M) [11].

The ACURATE Neo (Boston Scientific, Marlborough, MA) is a supra-annular self-expanding transcatheter heart valve at relatively low risk for conduction disorders [12]. The latest iteration, the Neo 2, includes a sealing fabric to mitigate paravalvular leaks. The project to look for early discharge in patients undergoing TAVI with the ACURATE (POLESTAR) study was a prospective observational study to evaluate safety and feasibility of ED within 48 h after TAVI with an ACURATE Neo valve in patients with symptomatic severe AS who were deemed candidates for ED prior to the TAVI procedure.

## Methods

POLESTAR was an international multi-center, prospective observational single-arm study (NCT03910751) conducted in the Netherlands, Belgium, the UK, and Canada. The rationale and design of the trial have been published previously [13]. Patients with symptomatic severe AS who were deemed suitable candidates for TAVI with ACURATE Neo valve and expected discharge to the individual’s home environment within 48 h were eligible for the study. Inclusion and exclusion criteria are reported in Supplementary Table 1. Key exclusion criteria were as follows: left ventricular ejection fraction (LVEF) < 35%, more than moderate mitral regurgitation, severe pulmonary hypertension (sPAP > 60 mmHg), unresolved complex coronary artery disease, presence of high-grade atrioventricular (AV) block or right bundle branch block, non-transfemoral access, severe peripheral artery disease, BMI > 35 kg/m^2^, frailty (determined by a multi-parametric assessment per local practice and separately discussed in the multidisciplinary heart team that may involve geriatricians), and inappropriate social support and/or familial care.

### Study procedures

Local multidisciplinary heart teams confirmed anatomical feasibility for transfemoral TAVI with ACURATE Neo based on multi-slice computed tomography and feasibility for early discharge based on the predefined set of inclusion and exclusion criteria. The intention for ED was discussed with all patients and the study team had to verify that proper social support was present should the patient be discharged early. Written informed consent was obtained prior to the TAVI procedure. The study was conducted in accordance with the principles of the Declaration of Helsinki and did not fall under the scope of the Medical Research Involving Human Subjects Act as determined by the Erasmus University Medical Center institutional ethics committee. Separate approval was given by the national research ethics committee of Belgium and the UK respectively. Site-specific research ethics boards provided approval in Canada.

Transfemoral TAVI was performed using ACURATE Neo or ACURATE Neo 2. Local anesthesia or conscious sedation, ultrasound-guided arterial access, and pacing on the left ventricle (LV) wire were recommended to streamline the TAVI procedure. Actual discharge timing was per treating physician’s discretion, based on advisory criteria and aligned with each individual patient. Patients were only eligible for early discharge if the QRS and AV intervals had plateaued or narrowed again within 48 h [13]. Transfer to a referring hospital or nursing care facility did not qualify as early discharge. All patients were contacted by telephone day + 1 and + 7 after discharge to assess serious adverse events.

### Endpoints

Clinical endpoints were according to the Valve Academic Research Consortium (VARC)-3 consensus [14]. The primary safety endpoint at 30 days was a composite of all-cause death, any stroke, VARC type 2–4 bleeding, acute kidney injury stage 3–4, major vascular, major access related and major cardiac structural complication, moderate or severe aortic regurgitation, new permanent pacemaker implantation (PPI), surgery, or intervention related to the transcatheter heart valve.

The efficacy endpoint was a composite of all-cause death, all stroke, rehospitalization for procedure- or valve-related causes, Kansas City Cardiomyopathy Questionnaire (KCCQ) Overall Summary Score (OSS) < 45, or decline from baseline > 10 points.

Secondary endpoints included early discharge success within 48 h after TAVI, occurrence of newly acquired conduction abnormalities, need for permanent pacemaker implantation, and prosthetic valve performance based on transthoracic echocardiography. Reasons for ED failure were recorded. Quality of life (QoL) was assessed at baseline and 30 days post-TAVI using the EQ-5D-5L index/utility score and the KCCQ.

### Statistics

Normality of continuous variables was tested using the Shapiro–Wilk test. Accordingly, continuous variables were described as mean with standard deviation, or as median with 25th and 75th percentile. Categorical variables were expressed as percentages and counts. The primary endpoints at 30 days were described as proportions. A landmark analysis of the efficacy and safety endpoints set at 2 days post-TAVI, the point at which ED was determined, with follow-up up to 30 days was performed using logistic regression with the discharge group as the independent variable and year of procedure as covariate. Odds ratios with 95% confidence interval were provided with the non-ED group as reference. Patients with a non-lethal event prior to the 2-day landmark were not excluded for the landmark analysis. Patients who died within 2 days after TAVI were excluded from the landmark analysis. EQ-5D-5L and KCCQ scores at baseline versus 30 days were compared using paired *t*-tests. All tests were two-tailed, and a *p*-value of < 0.05 was considered statistically significant.

An independent clinical research organization (Avania BV, Bilthoven, NL) assisted in monitoring at least 25% of source data. An independent clinical event committee adjudicated major adverse events. Boston Scientific provided an unrestricted grant but was not involved in data acquisition, analysis, or statistics. The first and last authors prepared the first draft of the manuscript. All co-authors reviewed and approved the manuscript.

## Results

Between April 2019 and December 2022, a total of 252 patients were included at 15 sites in the Netherlands, Belgium, Canada, and the UK. Baseline patient demographics are reported in Table 1. The median age was 82 [25th–75th percentile, 78–85] years, 53% were female, and the median Society of Thoracic Surgeons Predicted Risk of Mortality (STS-PROM) score was 2.2% [25th–75th percentile, 1.6–3.3]. Overall, 3936 transfemoral TAVI procedures were performed at the 15 participating sites during the study period.

**Table 1:** Baseline characteristics

	Overall *n* = 252	Early discharge *n* = 173	No early discharge *n* = 79	*p*-value
Age, years	82 [78–85]	82 [78–84]	82 [76–85]	0.40
Female	133 (53)	89 (51)	44 (56)	0.53
BMI, kg/m^2^	27 ± 3.9	27 ± 3.9	26 ± 3.8	< 0.01
Hypertension	148 (59)	104 (60)	44 (56)	0.48
Diabetes mellitus	54 (21)	41 (24)	13 (17)	0.19
eGFR < 60	90 (36)	64 (37)	26 (33)	0.53
Stroke or TIA	33 (13)	18 (10)	15 (19)	0.06
Peripheral artery disease	15 (6)	13 (8)	2 (3)	0.16
Myocardial infarction	21 (8)	14 (8)	7 (9)	0.84
PCI	62 (25)	40 (23)	22 (28)	0.42
CABG	23 (9)	19 (11)	4 (5)	0.16
Atrial fibrillation	46 (18)	27 (16)	19 (24)	0.11
Pacemaker/ICD	19 (8)	14 (8)	5 (6)	0.64
LBBB*	17 (8)	10 (7)	7 (10)	0.42
NYHA class^†^				0.65
I	14 (6)	8 (5)	6 (8)	
II	122 (49)	82 (48)	40 (51)	
III	113 (45)	80 (47)	33 (42)	
IV	-	-	-	
STS-PROM, %	2.2 [1.6–3.3]	2.3 [1.7–3.3]	2.2 [1.4–3.3]	0.64
Echocardiography				
LVEF, %	60 [55–62]	60 [55–63]	60 [55–62]	0.78
Peak gradient, mmHg	71 ± 20	71 ± 21	72 ± 19	0.69
Mean gradient, mmHg	43 ± 13	43 ± 14	43 ± 12	0.86
Aortic valve area, cm^2^	0.79 [0.64–0.90]	0.76 [0.61–0.90]	0.80 [0.70–0.90]	0.57
Aortic regurgitation ≥ moderate*	32 (13)	24 (14)	8 (11)	0.46
Mitral regurgitation ≥ moderate*	26 (11)	15 (9)	11 (14)	0.20
Tricuspid regurgitation ≥ moderate*	23 (9)	15 (9)	8 (11)	0.68

Values are numbers with (%), means ± SD, or medians with [25th–75th percentile]

*Percentages given of population without baseline pacemaker and with ECG available. ^†^Percentages given of non-missing population

*BMI* body mass index, *CABG* coronary artery bypass grafting, *COPD* chronic obstructive pulmonary disease, *eGFR* estimated glomerular filtration rate, *ICD* implantable cardioverter defibrillator, *LBBB* left bundle branch block, *LVEF* left ventricular ejection fraction, *PCI* percutaneous coronary intervention, *STS-PROM* Society of Thoracic Surgeons’ predicted risk of mortality, *TIA* transient ischemic attack

Procedural data are summarized in Table 2. An ACURATE Neo or Neo 2 was implanted in 98% of patients. Reasons for a different transcatheter platform than ACURATE were as follows: anatomical mismatch (*n* = 2), emergency surgery (*n* = 1), and no on-site availability of the proper ACURATE valve size (*n* = 1). In two patients, no clear reason was provided. Overall, two patients required conversion to surgery and three patients needed more than one transcatheter heart valve (due to two valve migrations, one severe aortic regurgitation [AR] due to high implant).

**Table 2:** Procedural characteristics

	Overall *n* = 252
Valve type	
- ACURATE Neo - ACURATE Neo 2 - Other	125 (50) 121 (48) 6 (2)
> 1 valve implanted	3 (1)
Concomitant PCI	10 (4)
Cerebral protection used	99 (39)
Local anesthesia Conscious sedation General anesthesia	153 (61) 97 (38) 2 (1)
Type arteriotomy closure*	
- Manta - Proglide/Prostar - Surgical Additional angioseal	48 (19) 200 (80) 1 (1) 59 (23)
Valve embolization	3 (1)^†^
Conversion to surgery	2 (1)^†^

Values are numbers with (%)

*In three patients, no type was reported; percentages are given of non-missing population. ^†^In one patient, valve embolization resulted in conversion to surgery

*PCI* percutaneous coronary intervention

Local anesthesia or conscious sedation was used in 153 (61%) and 97 (38%) patients respectively. Escalation to general anesthesia was required in the 2 patients (1%) because of conversion to surgery. A filter-based cerebral embolic protection device was used in 99 (39%) patients. Routine post arteriotomy closure angiography was performed in 9/15 sites. The other 6 sites made femoral angiography when clinically indicated.

**Fig. 1:**
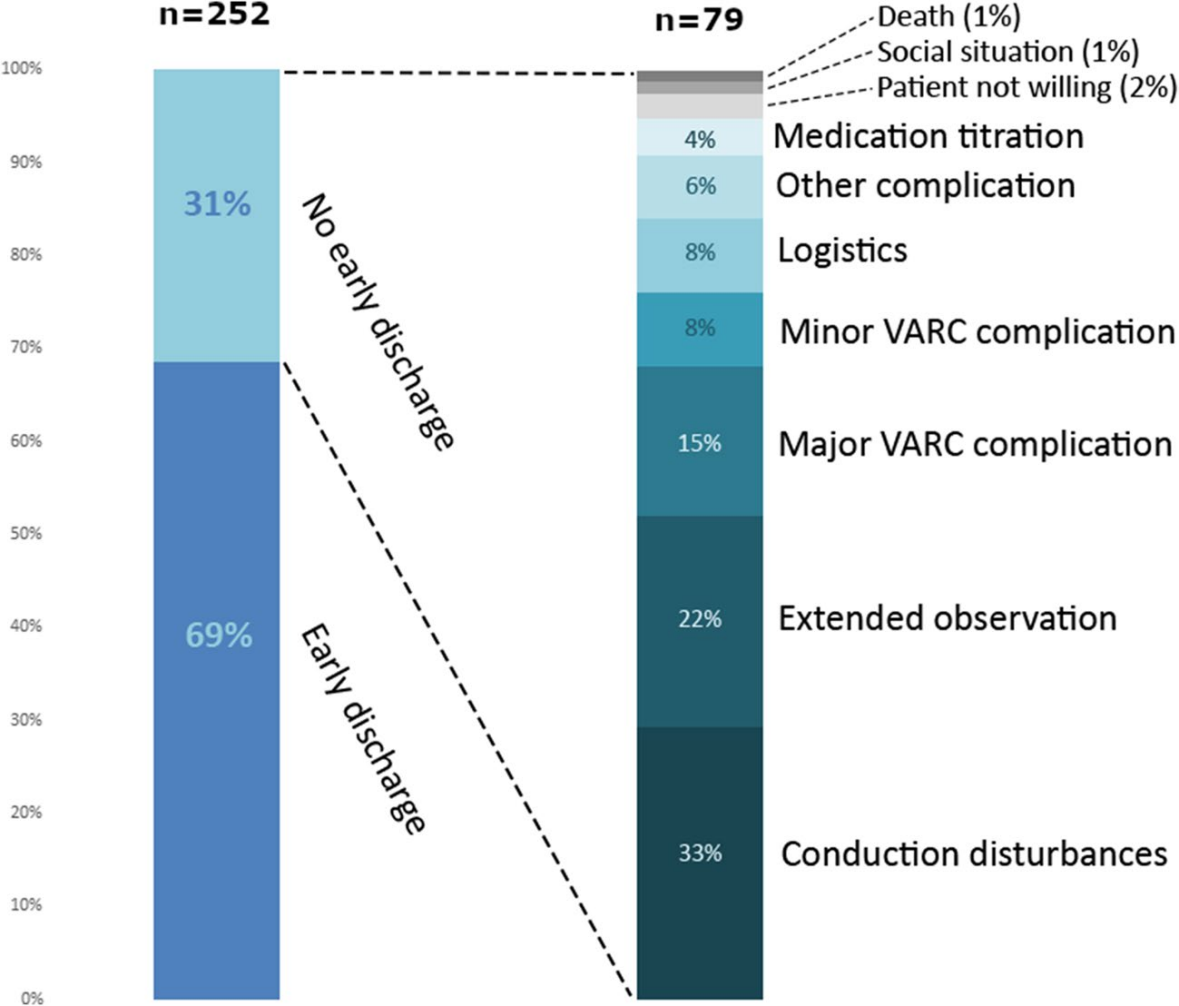
Reasons for no early discharge. Left histogram shows the total population. Right histogram shows the no early discharge subgroup. Complications are defined according to the Valve Academic Research Consortium (VARC)-3

Early discharge was achieved in 173 patients (69%), and in 79 patients (31%), discharge was delayed. Reasons for ED failure are tabulated in Fig. 1. Conduction disturbances with prolonged rhythm monitoring were needed in 33%, and extended (clinical) observation for the likes of fever and/or elevated CRP levels was reported in 22% and (major) VARC-defined complications in 15%. In the overall population, the median length of hospital stay was 2 [25th–75th percentile, 1–3] days. ED was more common in the 2nd half of the enrollment period (79/126 vs. 94/126, *p* = 0.04).

Compared to the ED group, patients with no ED had lower BMI and more often a history of previous stroke (Table 1). Regional differences were observed, with the highest ED rate in the UK and lowest in the Netherlands (Supplementary Table 2). After discharge, one patient withdrew from study participation.

Overall, all-cause mortality was 1% at 30 days with a stroke rate of 2%. Major vascular complications occurred in 4% of patients. A total of 34 (14%) patients reached a safety endpoint at 30 days. In the landmark analysis after 2 days, 7% and 9% of patients with ED and non-ED reached the safety endpoint respectively (OR 0.84 [25th–75th percentile, 0.31–2.26], *p* = 0.73) (Table 3). Clinical event rates decreased per year (OR 0.64 [25th–75th percentile, 0.41–0.99], *p* = 0.04). A total of 39 (16%) patients reached the efficacy endpoint at 30 days. The 2-day landmark analysis showed that 15% of patients with ED and non-ED reached the efficacy endpoint between 2 and 30 days (OR 0.97 [25th–75th percentile, 0.46–2.06], *p* = 0.94). Procedure year did not affect the efficacy endpoint (OR 0.86 [25th–75th percentile, 0.62–1.20], *p* = 0.38). No other covariates were included due to low number of events.

**Table 3:** Primary outcomes at 30 days

	Overall *n* = 251	Overall landmarked at 2 days *n* = 250	No early discharge vs. early discharge landmarked at 2 days		
			Early discharge	No early discharge	*p-*value
Safety endpoint	34 (13.5)	19 (7.6)	12 (7.0)	7 (9.0) OR 0.84 [0.31–2.26]	0.73
Efficacy endpoint	39 (15.5)	37 (14.9)	25 (14.5)	12 (15.4) OR 0.97 [0.46–2.06]	0.94

Values are numbers with (%) or odds ratios with [95% confidence intervals] and no early discharge as the reference group. The composite safety endpoint TAVI included the following: all-cause death, any stroke, VARC type 2–4 bleeding, acute kidney injury stage 3–4, major vascular, major access related and major cardiac structural complication, moderate or severe aortic regurgitation, new permanent pacemaker implantation, surgery, or intervention related to the transcatheter heart valve. The composite efficacy endpoint included all-cause death, all stroke, rehospitalization for procedure- or valve-related causes, KCCQ Overall Summary Score < 45, or decline from baseline > 10 points. One patient withdrew from follow-up directly after discharge. Odds ratios with [95% confidence intervals] are provided with no early discharge as the reference group

Secondary outcomes at 30 days are reported in Table 4 and Supplementary Table 3. Overall, 9 patients (4%) received new permanent pacemaker implantation (PPI). Reasons for PPI were total atrioventricular block (*n* = 7) or new left bundle branch block with increasing PR interval (*n* = 2). Of these patients, 6 required new PPI during the index hospitalization, all of which were discharged > 48 h after TAVI. Three (2%) patients in the ED group required PPI, which all occurred after discharge, and thus required rehospitalization. Of the 26 patients who had extensive telemetry monitoring beyond 48 h, 6 (23%) received PPI before discharge. Notably, there was no difference in permanent pacemaker, left bundle branch block, or atrial fibrillation between discharge groups at baseline.

**Table 4:** Secondary outcomes at 30 days

	Overall *n* = 251*	Early discharge *n* = 172	No early discharge *n* = 79	*p*-value
All-cause death	2 (1)	1 (1)	1 (1)	0.53
Cardiovascular death	2 (1)	1 (1)	1 (1)	0.53
Stroke	4 (2)	1 (1)	3 (4)	0.09
VARC 2–4 bleeding	8 (3)	2 (1)	6 (8)	0.01
Acute kidney injury stage 3–4	1 (1)	-	1 (1)	0.32
Major vascular complication	10 (4)	3 (2)	7 (9)	0.01
Major access related complication	1 (1)	-	1 (1)	0.32
Major cardiac structural complication	2 (1)	-	1 (1)	0.10
Moderate or severe AR^†^	7 (3)	6 (4)	1 (1)	0.43
New permanent pacemaker	9 (4)	3 (2)	6 (8)	0.03
New conduction disturbances^‡^, on discharge ECG	52 (21)	25 (15)	27 (34)	< 0.01
Surgery or intervention related to valve	2 (1)	-	2 (3)	0.10
All-cause rehospitalization	18 (7)	11 (6)	7 (9)	0.48
Rehospitalization for procedure or valve related cause	10 (4)	5 (3)	5 (6)	0.29
KCCQ OSS < 45 or decline > 10 points^§^	26 (11)	19 (12)	7 (10)	0.68
Endocarditis	2 (1)	1 (1)	1 (1)	0.53
Myocardial infarction	-	-	-	-

Values are numbers with (%)

^*^1 patient withdrew from follow-up directly after discharge, leading to *n* = 251 patients. ^†^Of 235 patients, determined between TAVI and 30 days. ^‡^New permanent bundle branch block, any new AV block, any new permanent pacemaker. ^§^Number of missing *n* = 21, percentages given of non-missing population

*AR* aortic regurgitation, *KCCQ OSS* Kansas City Cardiomyopathy Questionnaire Overall Summary Score, *VARC* Valve Academic Research Consortium

At 30 days, 18 (7%) patients were readmitted with 10/18 (56%) patients hospitalized for procedure- or valve-related reasons. No significant differences were observed between ED and non-ED groups in terms of all-cause rehospitalization and rehospitalization for procedure- or valve-related causes. (6% vs. 9%, *p* = 0.48 and 3% vs. 6%, *p* = 0.29, respectively).

Echocardiography-derived hemodynamic valve performance at 30 days showed a mean AVA of 2.0 ± 0.6 cm and residual mean gradient of 8 mmHg (6–12 mmHg). More than mild AR was present in 3% of patients (Supplementary Table 4). No difference was observed in > mild AR between ACURATE Neo and Neo 2 (3/114 vs. 4/115, *p* = 0.99).

The median EQ-5D-5L index score improved from 0.83 [0.72–0.91] at baseline to 0.88 [0.80–1.00] (*p* < 0.01) at 30 days. The KCCQ Overall Summary Score increased from 66 [50–85] to 87 [71–95] (*p* < 0.01) at 30 days (Supplementary Table 5). KCCQ improved similarly in patients with ED and no ED. EQ-5D-5L VAS improved more in the ED cohort (Supplementary Table 6). Data for TAVI with ACURATE Neo and Neo 2 are reported in Supplementary Table 7.

## Discussion

In the prospective POLESTAR trial, patients were selected for early discharge before TAVI with ACURATE NEO valve. The main findings were the following. [1] Two-thirds of patients were discharged within 48 h and one-third of patients more than 48 h after the TAVI procedure. [2] Prolonged rhythm monitoring for acquired conduction disorders was the main cause for delayed discharge. [3] Landmarked safety and efficacy endpoint event rates (between 2 and 30 days) were similar for patients with early and late discharge. [4] Readmission rate for patients who were discharged early was low (6%). [5] QoL after TAVI improved similarly in patients with early and delayed discharge.

In POLESTAR, standardized criteria were applied prior to the TAVI procedure to screen patient eligibility for early discharge to the respective home environment within 48 h [13]. In the prospective 3 M trial, 90% of patients were successfully discharged home within 48 h as compared to 51% in the FAST-TAVI trial [10, 11]. These differences should be interpreted on the background of fundamental differences in trial design. In FAST-TAVI, patients were only selected for early discharge after the TAVI procedure was (successfully) completed, which contrasts with POLESTAR that identified and enrolled patients prospectively before the TAVI procedure [10]. In 3 M, patients had to have a life expectancy of at least 3 years, which resulted in a more selective patient cohort at lower procedural risk [11]. More recently, same-day discharge after TF-TAVI was deemed feasible in 124 patients who were eligible based on predominantly baseline ECG criteria [15]. However, the study did not provide data on patients who were deemed eligible for early discharge prior to procedure and who were not subsequently discharged. The relative safety is therefore unknown.

Retrospective observational studies also looked at early discharge protocols. One study reported on clinical outcomes in a next-day discharge patient group in comparison with a delayed discharge group without in-hospital complications [16]. POLESTAR did not exclude patients with in-hospital complications (potentially avoiding bias) and showed relative safety in a landmark analysis. Furthermore, our trial provides insights in the reason for delayed discharge and shows that serious adverse events are only part of the explanation for extended hospital stay. Another retrospective study reported on patients who underwent same-day and next-day discharge and showed low PPI and readmission rates [17]. However, unlike POLESTAR, this study did not provide data on the population that was initially eligible for early discharge but was not discharged early.

We noticed geographical differences in terms of early discharge success in POLESTAR with lower success in the Netherlands and higher success in the UK. Dutch sites started with study enrollment earlier and may have still been in the process of refining the local early discharge protocols as opposed to UK sites that may have been more experienced with early discharge and already had dedicated clinical TAVI pathways in place. Also, COVID-19 may have affected TAVI practice and stimulated early discharge. Indeed, early discharge rates became higher in the second half of study enrollment (between October 2021 and December 2022) that overlapped with the COVID era. Contrasts in reimbursement between countries might have contributed to different early discharge rates and further research into causality is required.

The need for continued monitoring for acquired conduction disorders was the dominant reason for discharge after 48 h. TAVI with ACURATE is associated with low new pacemaker rates [18], and in POLESTAR, the overall pacemaker rate was only 4%. These rates might be explained by exclusion of patients with untreated high-degree conduction disorders as well as the specific transcatheter valve design that was used in the trial. Interestingly, most new pacemakers were implanted in patients who were discharged after 48 h because of prolonged rhythm monitoring. None of the patients who were monitored beyond 48 h and discharged without a new pacemaker required a permanent pacemaker after hospital discharge. Conversely, three patients (2%) who were discharged within 48 h were readmitted for a high-grade AV block, and received a new permanent pacemaker. This low rate of new pacemaker implantations after initial hospital discharge has been reported before, with a 2% readmission rate for PPI at 90 days in a large early discharge TAVI cohort [19]. In the same study, the authors report a 1.8% PPI readmission rate for the non-ED group which is higher than in POLESTAR (none). This attests to the rigor of postprocedural monitoring in our trial.

Patients in POLESTAR were octogenarians and may be considered at intermediate operative risk based on age and comorbidities [20–22]. There was no difference in the landmarked composite 30-day safety and efficacy endpoints between patients with and without early discharge. Notably, the safety endpoint was reached in 17 patients before 48 h and in 19 patients after 48 h. Of the 19 patients who reached the safety endpoint after 48 h, seven occurrences were due to moderate or severe AR that was revealed by pre-discharge TTE and did not result in any further clinical event. Overall, 7% of patients were readmitted after hospital discharge with no difference between patients with vs. without early discharge. This compares with the 9–10% all-cause readmission rate in the M3 and FAST-TAVI trials. Valve- or procedure-related rehospitalization was 4% in POLESTAR compared to 4% and 6% cardiac rehospitalization in FAST-TAVI and 3 M respectively. Low-risk RCTs reported readmission rates of 1 and 3% [23, 24].

As expected, we found more conduction disorders, major bleedings, major vascular complications, and new permanent pacemaker implantations in patients who were discharged beyond 48 h. All-cause rehospitalization and procedure- or valve-related rehospitalization did not differ between groups. TAVI resulted in the same meaningful improvements in quality of life for patients with and without early discharge. Baseline and follow-up QoL assessed with KCCQ and EQ-5D-5L scores showed similar results as in low-risk TAVI populations. ED patients showed more improvement on the visual analogue scale than no-ED patients. There was no difference in KCCQ OSS and EQ-5D-5L index scores between ED and non-ED cohorts.

## Limitations

The POLESTAR trial has several limitations. Decision for early discharge remained per treating physicians’ discretion and was prone to selection bias and confounders not recorded in this trial. Institutional practices may differ nationally and internationally. COVID-19 impacted TAVI practice and readmission policies. POLESTAR aimed to reflect contemporary clinical practice minimizing additional trial-specific activities. There were missing data for quality of life and echocardiography measurements. All valve performance data were site reported in the absence of an independent echo core laboratory.

## Conclusion

Early discharge after TAVI with the ACURATE Neo and Neo 2 valve is safe and feasible in selected patients. Rhythm monitoring and extended clinical observation protracted hospital stay leading to delayed discharge in approximately a third of patients.

## Impact on daily practice

In pre-procedurally selected patients undergoing TAVI with the ACURATE Neo valve, we found an early discharge rate of 69% and identified major factors that postpone discharge and prolonged clinical observation. Early discharge was safe and feasible in a selected group of patients without any penalty in terms of early hospital readmission for the early discharge group. An early discharge policy could increase hospital turnover and expand TAVI capacity while preserving patient safety.

## Supplementary Information

Below is the link to the electronic supplementary material.

Supplementary file 1 (DOCX 21 KB)
