# Correction: Safety and feasibility of early discharge after transcatheter aortic valve implantation with ACURATE Neo—the POLESTAR trial

**DOI:** 10.1007/s00392-026-02959-7

**Published:** 2026-06-08

**Authors:** Joris F. Ooms, Kristoff Cornelis, Harindra C. Wijeysundera, Bert Vandeloo, Jan Van Der Heyden, Jan Kovac, David Wood, Albert Chan, Joanna Wykrzykowska, Liesbeth Rosseel, Michael Cunnington, Frank van der Kley, Benno Rensing, Michiel Voskuil, David Hildick-Smith, Nicolas M. Van Mieghem

**Affiliations:** 1 Department of Interventional Cardiology, Thoraxcenter, Erasmus University Medical Center, Rotterdam, The Netherlands; 2 AZ Maria Middelares, Ghent, Belgium; 3 Sunnybrook Health Sciences Centre, Toronto, ON Canada; 4 University Hospital Brussels, Brussels, Belgium; 5 Sint Jan Hospital, Brugge, Belgium; 6 University Hospitals Leicester NHS Trust, Leicester, UK; 7 Centre for Heart Valve Innovation, St. Paul’s Hospital, University of British Columbia, Vancouver, BC Canada; 8 Royal Columbian Hospital, New Westminster, BC Canada; 9 University Medical Center Groningen, Groningen, The Netherlands; 10 ASZ Aalst, Aalst, Belgium; 11 Leeds Teaching Hospitals NHS Trust, Leeds, UK; 12 Leiden University Medical Center, Leiden, The Netherlands; 13 Sint Antonius Hospital, Nieuwegein, The Netherlands; 14 University Medical Center Utrecht, Utrecht, The Netherlands; 15 University Hospitals Brighton and Sussex NHS Trust, Brighton, UK

**Correction: Clinical Research in Cardiology (2024) 114:341-349**

10.1007/s00392-024-02436-z

When this article was first published, the name of the 9th author was mistakenly given as "Joanna Wykyrzykowska" though it is correctly "Joanna Wykrzykowska".

Also, the Electronic Supplementary Material mentioned in the text was not present.

The Original Article has been corrected.

