# Supplementary material for: Safety and feasibility of early discharge after transcatheter aortic valve implantation with ACURATE Neo—the POLESTAR trial
Source: Clin Res Cardiol. 2024 Apr 15;114(3):341–9. doi: 10.1007/s00392-024-02436-z (PMC11913897; doi:10.1007/s00392-024-02436-z)
Supplement: Supplementary file 1 — (DOCX 21 KB) [file 392_2024_2436_MOESM1_ESM.docx]

**Supplementary Tables**

| **S1. Inclusion Criteria** |
| --- |
| Age ≥ 18 years |
| Eligible for transfemoral Transcatheter Aortic Valve Implantation with the ACURATE Neo platform |
| Able to provide written informed consent |
| **Exclusion Criteria** |
| **Cardiac** |
| Left ventricular ejection fraction <35% |
| More than moderate mitral regurgitation |
| Severe pulmonary hypertension (sPAP >60mmHg) |
| Unresolved complex coronary artery disease *defined as: multivessel disease, left main lesions, bifurcation lesions* |
| Untreated high degree atrioventricular block or right bundle branch block |
| **Other comorbidities/conditions** |
| Body Mass Index > 35 kg/m^2^ |
| Pregnancy |
| Chronic Obstructive Pulmonary Disease class > II |
| Chronic kidney dysfunction (eGFR < 35ml/min) |
| Frailty per multi-disciplinary heart team evaluation |
| Walking aid dependent for mobilization |
| Inappropriate social support and/or familial care |
| **TAVI strategy** |
| Severe peripheral artery disease (History) *defined as: claudication within <200m, previous thromboendarterectomy, plastic patch in situ of common femoral artery, uni-/bilateral lower extremity amputation*  Severe peripheral artery disease (Computed Tomography)  *defined as: protrusion of calcium into the arterial lumen and/or circumferential distribution at the level of intended access site* |
| Non-transfemoral access TAVI |
| **Follow-up** |
| Inability to adhere to planned follow-up of one year |

| **S2 - Geography** | **Early discharge (%)** | **Composite of death and all-cause rehospitalization at 30 days (%)** | **Median KCCQ OSS change at 30 days [25^th^-75^th^ percentile]** |
| --- | --- | --- | --- |
| Netherlands | 77/130 (59) | 8/130 (6) | 13 [2-27] |
| Belgium | 33/51 (65) | 6/51 (12) | 10 [0-27] |
| Canada | 34/39 (87) | 4/39 (10) | 5 [-1-20] |
| United Kingdom | 29/32 (91) | 2/31 (6) | 21 [7-40] |
| *p-value* | <0.01 | 0.58 | 0.07 |

| **S3. Secondary outcomes at 30 days** | **Prior to landmark – 48hrs** | | | **After landmark – 48hrs** | | |
| --- | --- | --- | --- | --- | --- | --- |
|  | Early discharge  n=173 | No early discharge  n=79 | *p-value* | Early discharge  n=172 | No early discharge  n=78 | *p-value* |
| All-cause death | - | 1 (1) | 0.31 | 1 (1) | - | 0.99 |
| Cardiovascular death | - | 1 (1) | 0.31 | 1 (1) | - | 0.99 |
| Stroke | - | 2 (3) | 0.10 | 1 (1) | 1 (1) | 0.53 |
| VARC 2-4 bleeding | - | 6 (8) | <0.01 | 2 (1) | - | 0.99 |
| AKI stage 3-4 | - | 1 (1) | 0.31 | - | - | - |
| Major vascular* | 2 (1) | 5 (6) | 0.03 | 1 (1) | 3 (4) | 0.10 |
| Major access related | - | - | - | - | 1 (1) | 0.32 |
| Major cardiac structural | - | 2 (3) | 0.10 | - | - | - |
| New PPI | - | 4 (5) | 0.01 | 3 (2) | 2 (3) | 0.65 |
| Surgery or intervention related to THV | - | 2 (3) | 0.10 | - | - | - |

Values are numbers with (%). *One patient had 2 major vascular events, one before landmark, one after landmark. Therefore, here a total of 11 major vascular events are reported whereas 10 are reported at 30 days.

| **S4 - Prosthetic valve performance on echocardiography** | **Overall** | **Early discharge** | **No early discharge** | *p-value* |
| --- | --- | --- | --- | --- |
| Peak gradient (n=226) | 16 [12-22] | 16 [12-22] | 16 [12-23] | 0.73 |
| Mean gradient (n=221) | 8 [6-12] | 8 [5-11] | 9 [6-12] | 0.17 |
| Aortic valve area (n=149) | 2.0 ± 0.6 | 2.0 ± 0.5 | 2.1 ± 0.6 | 0.35 |
| Aortic regurgitation (of n=235)   - <mild - mild - moderate - severe | 120 (51) 108 (46) 7 (3) - | 80 (51)  71 (45)  6 (4)  - | 40 (51)  37 (47)  1 (1)  - | 0.55 |

Values are numbers with (%), means ±SD or medians with [25th-75th percentile]. Echocardiograms were obtained within 30-days post TAVI. Number of patients with data available were 226, 221, 149 and 235 for peak gradient, mean gradient, aortic valve area and aortic regurgitation respectively.

| **S5. Quality of life scores of overall population** | **Baseline** | **30 Days** | P-value |
| --- | --- | --- | --- |
| EQ-5D-5L index score  Score difference | 0.83 [0.72-0.91]  - | 0.88 [0.80-1.00] 0.04 [0-0.15] | <0.001 |
| EQ-5D-5L VAS  Score difference | 70 [60-80]  - | 80 [70-85] 5 [-3-15] | <0.001 |
| KCCQ Clinical summary score | 74 [60-88] | 89 [78-96] | <0.001 |
| Clinical summary score difference  Dead**  Worse (<-5)  No change (-5 and <5)  Mild improvement (5 and <10)  Moderate improvement (10 and <20)  Substantially improvement (≥20) | - | 8 [0-23]  2 (1) 29 (13) 63 (27) 33 (14) 40 (17)  65 (28) |  |
| KCCQ Overall summary score | 66 [50-85] | 87 [71-95] | <0.001 |
| Overall summary score difference  Dead**  Worse (<-5)  No change (-5 and <5)  Mild improvement (5 and <10)  Moderate improvement (10 and <20)  Substantially improvement (≥20 | - | 12 [2-27]  2 (1) 27 (11) 51 (22)  31 (13) 41 (18)  80 (35) |  |

Numbers are reported as

EQ5DL-Index score: 100 = maximal health. Baseline missing: VAS (5), EQ-5D-5L (4), KCCQ (3)

30 Day missing: VAS (22), EQ-5D-5L (20), KCCQ (19). **percentages are given of non-missing population. Of note, a change in 5 points is small but clinically important, 10-20 moderate to large (Spertus JACC 2020).

| **S6. Quality of life scores of discharge groups – differences at 30 days** | **Early discharge** | **No early discharge** | *p-value* |
| --- | --- | --- | --- |
| EQ-5D-5L index score difference | 0.04 [0-0.15] | 0.03 [-0.06-0.14] | 0.38 |
| EQ-5D-5L VAS difference | 5 [0-15] | 0 [-5-10] | 0.02 |
| KCCQ Clinical summary score (Css) difference  Dead**  Worse (<-5)  No change (-5 and <5)  Mild improvement (5 and <10)  Moderate improvement (10 and <20)  Substantially improvement (≥20 | 8 [0-22]  1 (1)  20 (12)  44 (27)  22 (14)  33 (21)  41 (26) | 8 [0-24]  1 (1)  9 (13)  19 (27)  11 (16)  7 (10)  24 (34) | 0.99  0.42 |
| KCCQ Overall summary score (Oss) difference  Dead**  Worse (<-5)  No change (-5 and <5)  Mild improvement (5 and <10)  Moderate improvement (10 and <20)  Substantially improvement (≥20 | 12 [2-28]  1 (1)  16 (10)  38 (24)  22 (14)  28 (17)  56 (35) | 12 [1-26]  1 (1)  11 (16)  13 (18)  9 (13)  13 (18)  24 (34) | 0.57  0.80 |

Values are numbers with (%) or medians with [25th-75th percentile]. *Percentages are given of non-missing population. VAS: visual analogue scale. KCCQ difference was missing in ED 13/173, no ED 9/79 (p=0.31). EQ5D difference was missing in ED 14/173, no ED 10/79 (p=0.25). EQ5D-VAS difference was missing in ED 17/173, no ED 10/79 (p=0.50).

| **S7 - Clinical outcomes at 30 days by valve** | **ACURATE NEO**  **(125)** | **ACURATE NEO 2 (120)*** | *p-value* |
| --- | --- | --- | --- |
| All-cause death | 1 (1) | 1 (1) | 0.99 |
| Cardiovascular death | 1 (1) | 1 (1) | 0.99 |
| Stroke | 1 (1) | 3 (3) | 0.36 |
| VARC 2-4 bleeding | 7 (6) | 1 (1) | 0.07 |
| AKI stage 3-4 | 1 (1) | - | 0.99 |
| Major vascular | 4 (3) | 5 (4) | 0.75 |
| Major access related | - | 1 (1) | 0.49 |
| Major cardiac structural | 1 (1) | - | 0.99 |
| Aortic regurgitation on TTE^§^  <Mild  Mild  Moderate Severe | 54/114  57/114  3/114  - | 63/115  48/115  4/115  - | 0.45  - |
| New PPI | 4 (3) | 5 (4) | 0.75 |
| New conduction disturbances^§§^ - On discharge ECG | 20 (16) | 28 (23) | 0.16 |
| Surgery or intervention related to THV | 1 (1) | - | 0.99 |
| All-cause Rehospitalization  Rehospitalization for procedure or valve related cause | 8 (6)  4 (3) | 9 (8)  5 (4) | 0.81  0.74 |
| KCCQ Oss <45 or decline >10points** | 13 (11) | 13 (12) | 0.99 |

Values are numbers with (%). *1 Patient was lost to follow-up, therefore 120 instead of 121

^§^Of 229 patients, determined between TAVI and 30-days

^§§^new permanent BBB, any new AV-block, any new permanent pacemaker

**Percentage of non-missing (n=116 and n=110 for Neo and Neo2 respectively)
